# ﻿Molecular evidence and differences in gonopod morphology lead to the recognition of a new species of the freshwater crab genus *Candidiopotamon* Bott, 1967 (Crustacea, Brachyura, Potamidae) from eastern Taiwan

**DOI:** 10.3897/zookeys.1179.106718

**Published:** 2023-09-11

**Authors:** Hsi-Te Shih, Tohru Naruse, Christoph D. Schubart

**Affiliations:** 1 Department of Life Science and Global Change Biology Research Center, National Chung Hsing University, 250, Kuo Kuang Road, Taichung 402, Taiwan National Chung Hsing University Taichung Taiwan; 2 Tropical Biosphere Research Center, Iriomote Station, University of the Ryukyus, 870 Uehara, Taketomi, Okinawa 907-1541, Japan University of the Ryukyus Okinawa Japan; 3 Zoology & Evolution, University of Regensburg, 93040 Regensburg, Germany University of Regensburg Regensburg Germany

**Keywords:** 16S rDNA, *Candidiopotamonpenglai*, *Candidiopotamonrathbuni*, cytochrome oxidase subunit I, Decapoda, morphology, new species, taxonomy

## Abstract

A new freshwater crab of the potamid genus *Candidiopotamon* Bott, 1967, is described from eastern Taiwan. *Candidiopotamonpenglai* sp. nov. is morphologically similar to *C.rathbuni* (De Man, 1914) from western Taiwan, but can be distinguished by the morphology of the male first gonopod (G1), as well as by their mitochondrial DNA (16S rRNA and COI genes). In the G1 of *C.rathbuni*, the subterminal segment shows a cline from robust in northern populations to slender in southern populations. In the G1 of *C.penglai***sp. nov.**, a distinctly larger and more distally directed keel-like projection is found on the distal inner edge of the terminal segment, with northern populations having an inward-curving subterminal segment and southern populations a straight subterminal segment. The genetic differentiation of the two species of *Candidiopotamon* within Taiwan is discussed, and morphological differences are compared. A key to the species of *Candidiopotamon* is also provided.

## ﻿Introduction

Taiwanese freshwater systems are populated by three genera of potamid freshwater crabs, viz., *Candidiopotamon* Bott, 1967, *Geothelphusa* Stimpson, 1858, and *Nanhaipotamon* Bott, 1968, with the former two often occurring in sympatry. Recent molecular studies suggested that the colonization of *Candidiopotamon* and *Geothelphusa* to Taiwan took place shortly after Taiwan was geologically shaped as an island, ca 5–6 mya (Shih et al. 2006, 2011). Subsequent geological uplifts of mountain chains have resulted in parallel separation processes in both genera of crabs, with similar time estimates based on mitochondrial DNA (Shih et al. 2004, 2006, 2007, 2011).

Despite sharing the same evolutionary history and the same habitat, the taxonomic situation of these two genera is quite different. In the case of *Geothelphusa*, nearly 40 species have been described from Taiwan, many of them recently (Shih and Ng 2011; Shy et al. 2014, 2020, 2021), and new species descriptions are still underway (cf. Shy et al. 2020), although a few of them have been synonymized (Shih and Shy 2009; Shih and Ng 2011; Shy et al. 2020, 2021) or at least suggested to be conspecific (Shih et al. 2023). In contrast, *Candidiopotamon* has only one described species in Taiwan, i.e., *Candidiopotamonrathbuni* (De Man, 1914), occurring throughout the island. Shih et al. (2006), however, showed that *C.rathbuni* in Taiwan is not homogeneous and consists of several (5 or 6) evolutionary significant units (ESU sensu Ryder 1986; Waples 1991; Chu et al. 2015) that can be identified genetically, appear to be reproductively isolated, geographically defined, and whose evolution can be explained by tectonic events that date back hundreds of thousands to several million years.

In the present study, we describe a new pseudocryptic species, *C.penglai* sp. nov., for the eastern populations of Taiwanese *Candidiopotamon*. We also conduct detailed morphological examinations, and molecular analyses using 16S rDNA and cytochrome oxidase subunit I (COI), with additional samples of Taiwanese representatives of *Candidiopotamon*, to test whether external characters related to reproduction correspond to genetic differences. The two Taiwanese species of *Candidiopotamon* are also compared with three congeners from the Ryukyu Islands in Japan, viz., *C.kumejimense* Minei, 1973, *C.okinawense* Minei, 1973, and *C.tokashikense* Naruse, Segawa & Aotsuka, 2007.

## ﻿Materials and methods

### ﻿Taxon sampling and morphological characters

**Figure 1.**
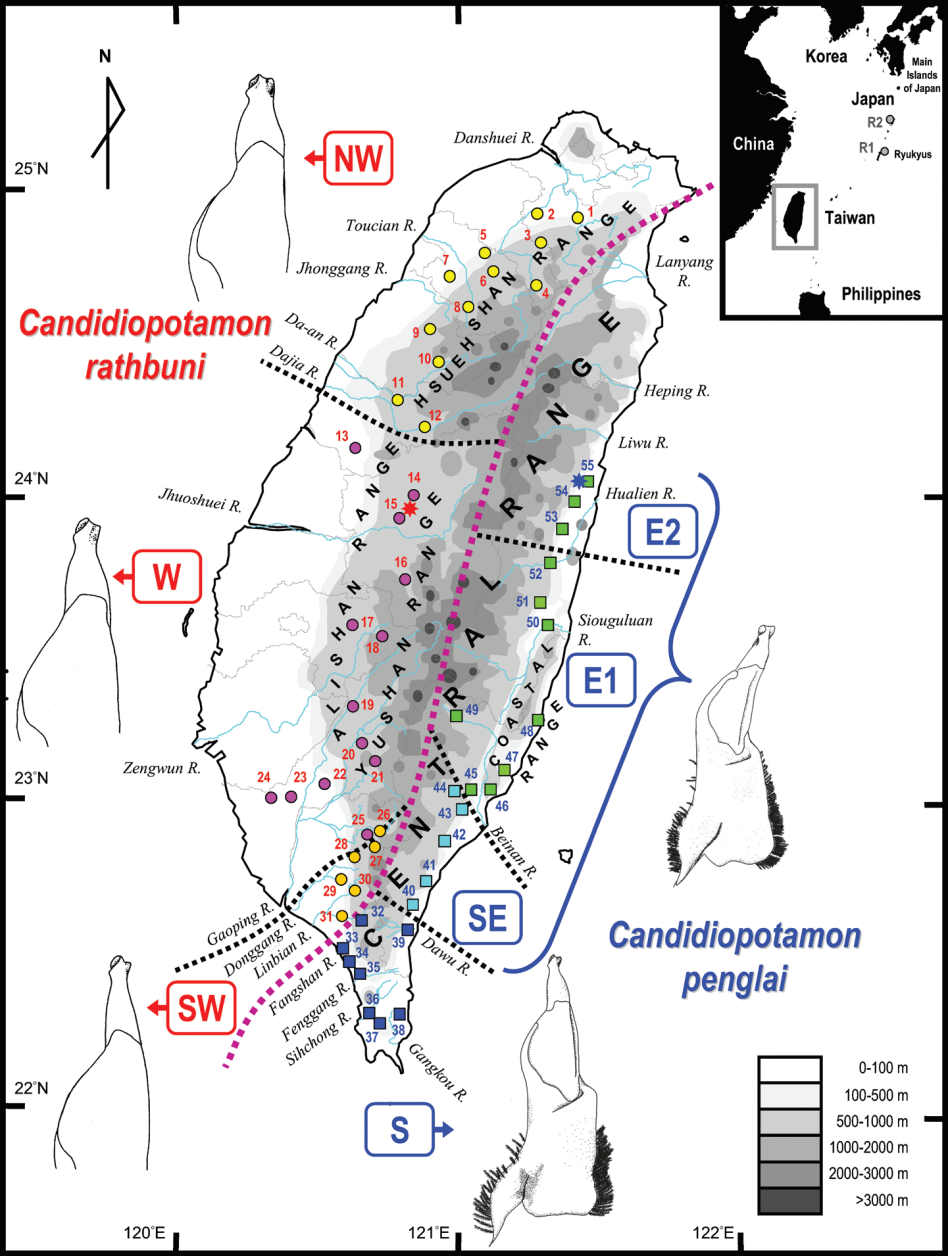
Collection sites for *Candidiopotamonrathbuni* (De Man, 1914) (circles) and *Candidiopotamonpenglai* sp. nov. (squares) in Taiwan, as well as other congeners from the Ryukyus, Japan. Different colors indicate sampling sites of clades. Dotted lines indicate the possible biogeographic boundaries between clades NW, W, SW, S, SE, E1, and E2, corresponding to the color in Fig. 2; as well as species. The red star and blue star indicate the type localities of *C.rathbuni* and *C.penglai* sp. nov., respectively. Three forms of male left G1 are shown for clades in *C.rathbuni* and two forms for clades in *C.penglai* sp. nov.

Specimens of the Taiwanese *Candidiopotamon* species were collected from mountain streams and coastal plains (Fig. 1, Table 1) and were preserved in 75–95% ethanol. Specimens of the following congeners were used as comparative material: *Candidiopotamonkumejimense* Minei, 1973: 1 male, 32.6 × 36.5 mm, RUMF-ZC-2596, Gima River, Kumejima Island, Ryukyu Islands, Japan, coll. Y. Fujita, 6 Aug. 2010. *Candidiopotamonokinawense* Minei, 1973: 1 male, 37.6 × 41.6 mm, RUMF-ZC-547, Mt. Nishime, Okinawa Island, Ryukyu Islands, Japan, coll. N. Kawauchi, 20 Jun. 1999. *Candidiopotamontokashikense* Naruse, Segawa & Aotsuka, 2007: 1 male, 36.4 × 41.7 mm, RUMF-ZC-220, stream on southeastern side of mountain located between Tokashiki and Tokashiku, Tokashiki Island, Ryukyu Islands, Japan, coll. Ryoko D. Segawa and T. Aotsuka, 15 Apr. 1997.

**Table 1.** Haplotypes of 16S and COI of *Candidiopotamonrathbuni* (De Man, 1914) and *Candidiopotamonpenglai* sp. nov. collected from different populations of Taiwan, as well as the outgroups from the Ryukyus. Numbers within square brackets correspond to localities in Fig. 1. R., river; Co., county.

Species and clades	Localities	Catalog no. of NCHUZOOL (unless indicated)	Sample size	16S	Access. nos.	COI	Access. nos.
***C.rathbuni***							
**NW clade**							
	New Taipei City (Wulai [1])	13146	1	NW1	AB208590	NW1-C	AB625764
	New Taipei City (Wulai [1])	15189	1	NW2	AB208589	NW2-C	OR344947
	New Taipei City (Sansia [2])	12956	2	NW3	OR346841, OR346842	NW3-C	OR344948, OR344949
	New Taipei City (Wulai [1]); Hsinchu Co. (Guansi [5]; Jianshih [6]; Wufong [8])	13146; 12927; 12915; 12914	5	NW4	AB208591, AB208591, AB208591, AB208591, AB208591	NW4-C1	OR344950, OR344951, AB433579, OR344952, OR344953
	Taoyuan (Dongyanshan, Fusing [3])	12955	2	NW4	OR346843, OR346844	NW4-C2	OR344954, OR344955
	Taoyuan (Baling, Fusing [4])	12954	1	NW4	OR346845	NW4-C3	OR344956
	Hsinchu Co. (Beipu [7]); Miaoli (Baguali, Tai-an [9])	12917; 12916	2	NW4	AB208591, AB208591	NW4-C4	OR344957, OR344958
	Hsinchu Co. (Beipu [7])	12917	1	NW4	AB208591	NW4-C5	OR344959
	Miaoli (Erbensong [10])	15193	1	NW4	AB208591	NW4-C6	OR344960
	Hsinchu Co. (Jianshih [6])	12904	1	NW5	AB208592	NW5-C	OR344961
	Miaoli (Baguali, Tai-an [9])	12916	1	NW6	AB208593	NW4-6	OR344962
	Taichung (Dongshih [11])	12918	1	NW7	AB208594	NW7-C1	OR344963
	Taichung (Dongshih [11])	12918	1	NW7	AB208595	NW7-C2	OR344964
	Taichung (Dongshih [11)	12918	1	NW7	AB208596	NW7-C3	OR344965
	Taichung (Guguan [12])	12941	1	NW8	AB208597	NW8-C	OR344966
**W clade**							
	Taichung (Caohu, Dali [13]; Taiping [13]); Nantou (Shueili [15])	12944; 12924; 12902	3	W1	AB208598, AB208598	W1-C	OR344967, OR344968, OR344969
	Nantou (Shueili [15])	17185	2	W2	OR346846, OR346847	W2-C	OR344970, OR344971
	Nantou (Shueili [15])	12902	1	W3	AB208599	W3-C1	OR344972
	Nantou (Lianhua Pond, Yuchih [14])	12903	1	W3	AB208599	W3-C2	OR344973
	Nantou (Lianhua Pond, Yuchih [14])	12903	1	W3	AB208599	W3-C3	OR344974
	Nantou (Shueili [15])	15187	1	W4	AB208600	W4-C	OR344975
	Nantou (Yapingshan, Sinyi [16])	12393	1	W5	OR346848	W5-C	OR344976
	Chiayi Co. (Guanhua, Jhuci [17])	12942	1	W6	AB208601	W6-C1	OR344977
	Chiayi Co. (Dabang, Alishan [18])	13609	1	W6	AB208601	W6-C2	OR344978
	Kaohsiung (Mincyuan, Sanmin [19])	12921	2	W6	AB208601, AB208601	W6-C3	OR344979, OR344980
	Kaohsiung (Baolai, Liouguei [20])	12920	1	W6	AB208601	W6-C4	OR344981
	Kaohsiung (Baolai, Liouguei [20])	12920	1	W6	AB208601	W6-C5	OR344982
	Kaohsiung (Daganshan, Alian [24])	12919	2	W6	AB208601, AB208601	W6-C6	OR344983, OR344984
	Kaohsiung (Tianliao [23])	12923	1	W6	AB208601	W6-C7	OR344985
	Kaohsiung (Tianliao [23])	12923	1	W6	AB208601	W6-C8	OR344986
	Kaohsiung (Chuyunshan, Taoyuan [21])	12922	1	W7	AB208602	W7-C	OR344987
	Kaohsiung (Meinong [22])	13368	1	W8	AB208603	W8-C	OR344988
	Pingtung (Ila, Wutai [25]; Jiamu, Wutai [25])	12928; 12949	2	W9	AB208604	W9-C	OR344989, OR344990
**SW clade**							
	Pingtung (Wutai [26])	12945	1	SW1	AB208607	SW1-C1	OR344991
	Pingtung (Haocha, Sandimen [27])	12947	1	SW1	AB208607	SW1-C2	OR344992
	Pingtung (Haocha, Sandimen [27])	15201	1	SW2	AB208606	SW2-C	OR344993
	Pingtung (Haocha, Sandimen [27])	15200	1	SW3	AB208608	SW3-C1	OR344994
	Pingtung (Liangshan, Majia [28])	17184	1	SW3	OR346849	SW3-C2	OR344995
	Pingtung (Taiwu [29])	12907	1	SW4	AB208605	SW4-C1	OR344996
	Pingtung (Taiwu [29])	15219	1	SW4	AB208605	SW4-C2	OR344997
	Pingtung (Taiwu [29])	15219	1	SW4	AB208605	SW4-C3	OR344998
	Pingtung (Laiyi [30])	12925	3	SW4	AB208605, AB208605, AB208605	SW4-C4	OR344999, OR345000, OR345001
	Pingtung (Lili, Chunrih [31])	12908	1	SW4	AB208605	SW4-C5	OR345002
***C.penglai***							
**S clade**							
	Pingtung (Jioucijia, Chunrih [32])	13074	1	S1	AB208609	S1-C1	OR345003
	Pingtung (Jioucijia, Chunrih [32])	13074	1	S1	AB208609	S1-C2	AB290649
	Pingtung (Nanshihhu R., Shihzih [33]; Cili R., Shihzih [33])	12939; 12948	2	S1	AB208610, AB208609	S1-C3	OR345005, OR345006
	Pingtung (Fangshan R., Shihzih [34])	12910	1	S1	AB208611	S1-C4	OR345007
	Pingtung (Maozaikengnei, Hengchun [37])	RUMF-ZC-57	1	S1	AB208615	S1-C5	OR345008
	Pingtung (Nanrenshan, Manjhou [38])	12940	1	S1	AB208615	S1-C6	OR345009
	Taitung (Dawu [39])	15220; 12943	2	S1	AB208616, AB208616	S1-C7	OR345010, OR345011
	Pingtung (Jioucijia, Chunrih [32])	13074	1	S2	AB208613	S2-C3	OR345012
	Pingtung (Fangshan R., Shihzih [34])	12910	1	S3	AB208612	S1-C3	OR345013
	Pingtung (Nanshihhu R., Shihzih [33])	12939	1	S4	AB208614	S4-C1	OR345014
	Pingtung (Danlu, Shihzih [35])	12912	1	S4	AB208614	S4-C2	OR345015
	Pingtung (Danlu, Shihzih [35])	15207	1	S4	AB208614	S4-C3	OR345016
	Pingtung (Sihchong R., Checheng [36])	NCHUZOOL	1	S4	AB208614	S4-C4	OR345017
	Pingtung (Nanrenshan, Manjhou [38])	NCHUZOOL	1	S4	AB208614	S4-C5	OR345018
	Pingtung (Nanrenshan, Manjhou [38])	15215	1	S4	OR346850	S4-C6	OR345019
**SE clade**							
	Taitung (Taiban, Daren [40])	NCHUZOOL	1	SE1	AB208617	SE1-C1	OR345020
	Taitung (Taiban, Daren [40])	15218	1	SE1	AB208617	SE1-C2	AB551394
	Taitung (Taiban, Daren [40])	15218	1	SE1	AB208617	SE1-C3	OR345022
	Taitung (Jhihben [42]; Taimali [41])	12929; 12931	2	SE1	AB208617, AB208617	SE1-C4	OR345023, OR345024
	Taitung (Taimali [41])	12953	2	SE1	OR346851, OR346852	SE1-C5	OR345025, OR345026
	Taitung (Jhihben [42])	12929	1	SE1	AB208617	SE1-C6	OR345027
	Taitung (Lijia, Beinan [43])	15160	1	SE1	AB208617	SE1-C7	OR345028
	Taitung (Lijia, Beinan [43])	12933	1	SE1	AB208617	SE1-C8	OR345029
	Taitung (Jhihben [42]	12986	1	SE2	OR346853	SE2-C	OR345030
	Taitung (Taimali) [41]	12931	1	SE3	AB208618	SE3-C1	OR345031
	Taitung (Taimali) [41]	12931	1	SE3	AB208618	SE3-C2	OR345032
	Taitung (Taimali) [41]	12931	1	SE3	AB208618	SE3-C3	OR345033
	Taitung (Taimali) [41]	12931	1	SE3	AB208618	SE3-C4	OR345034
	Taitung (Hongye, Yanping [44])	12937	1	SE4	AB208619	SE4-C	OR345035
	Taitung (Hongye, Yanping [44])	12937	1	SE5	AB208620	SE5-C	OR345036
	Taitung (Hongye, Yanping [44])	12937	1	SE6	AB208621	SE6-C	OR345037
**E1 clade**							
	Taitung (Luming R., Yanping [45]); Hualien (Jingpu, Fengbin [50])	12934; 12909	3	E1	AB208626, AB208626, AB208622	E1-C1	OR345038, OR345039, OR345040
	Taitung (Taiyuan, Donghe [48])	12936	1	E1	AB208622	E1-C3	OR345041
	Taitung (Luanshan, Yanping [47])	15212	1	E1	AB208624	E1-C4	OR345042
	Taitung (Chenggong, Sansian [49])	17183	1	E1	OR346854	E1-C5	OR345043
	Taitung (Jiafeng, Beinan [45])	NCHUZOOL	1	E2	AB208625	E2-C	OR345044
	Taitung (Chenggong, Sansian [49])	15206	1	E3	AB208623	E3-C	OR345045
	Taitung (Wulu, Haiduan [50])	12950	1	E4	OR346855	E4-C	OR345046
	Hualien (Fuyuan, Rueishuei [51])	12935	1	E1	AB208622	E1-C2	OR345047
	Hualien (Jili Lake, Guangfu [52])	15228	1	E1	OR346856	E1-C6	OR345048
	Hualien (Jili Lake, Guangfu [52])	15227	1	E1	OR346857	E1-C7	OR345049
**E2 clade**							
	Hualien (Shueiyuandi, Shoufeng [53]; Liyu Lake, Shoufeng [54]; Ji-an [55])	RUMF-ZC-8165 (paratype); 12952 (paratype); 15213 (paratype); 12932 (paratype); RUMF-ZC-8164 (paratype); 12951 (holotype)	6	E5	OR346858, OR346859, OR346860, OR346861, OR346862, OR346863, OR346864	E4-C	OR345050, OR345051, OR345052, OR345053, OR345054, OR345055, OR345056
All localities			112				
**Outgroups**							
*C.okinawense*	Ryukyus, Japan (Okinawa [R1])		1	Co	AB208627	Co-C	OR345057
*Amamikuamamense* Naruse, Segawa & Shokita, 2004	Ryukyus, Japan (Amami [R2])		1	Aa	AB428457	Aa-C1	OR345058
	Ryukyus, Japan (Amami [R2])		1	Aa	OR346865	Aa-C2	OR345059

We used the following abbreviations for morphological description: **CW**, carapace width; **CL**, carapace length; **G1**, male first gonopod; and **G2**, male second gonopod. The terminology follows Ng (1988), Dai (1999), Naruse et al. (2007), and Davie et al. (2015). Characters of G1 and G2 were measured using an eyepiece micrometer and a stereomicroscope (Nikon SMZ-10). To reduce the effect of allometric growth on measurements, only adult specimens were used for ratio characters. In the present study, minimum adult sizes were defined by estimating the inflection point of the major chela’s height for males; and by searching for the smallest individual whose pleonal 5^th^ somite width was equal to or wider than the 3^rd^ somite width for females. As a result, puberty sizes were provisionally defined as follows: *C.rathbuni*, male, 25.0 mm CW or 21.8 mm CL, female, 25.0 mm CW or 23.8 mm CL; *C.penglai*, male, 28.2 mm CW or 23.3 mm CL, female, 27.6 mm CW or 23.5 mm CL. Specimens examined are deposited in the Department of Life Science, National Chung Hsing University, Taiwan (**NCHUZOOL**), the National Science Museum, Tokyo, Japan (**NSMT**), the Ryukyu University Museum, Fujukan, Japan (**RUMF**), and the Zoological Reference Collection, the Lee Kong Chian Natural History Museum, National University of Singapore (**ZRC**).

### ﻿Genetic analyses

Genomic DNA was isolated from the muscle tissue of the legs by using the Sigma mammalian genomic DNA miniprep kit (Sigma-Aldrich, St. Louis, MO, USA) or the GeneMark tissue and cell genomic DNA purification kit (Taichung, Taiwan). A region of approximately 510 to 550 basepairs (bp) of the 5’-end of the mitochondrial large ribosomal subunit (16S rRNA) gene was selected for amplification with polymerase chain reaction (PCR) using the primers 1471, 1472 (Crandall and Fitzpatrick 1996), 16Sar, 16Sbr (Palumbi et al. 1991), 16L29 and 16H10 (Schubart 2009). A portion of the mitochondrial cytochrome oxidase subunit I (COI) gene was amplified with the primers LCO1490 and HCO2198 (Folmer et al. 1994), and LCOB (5’-CAAAYCATAAAGAYATYGG-3’), HCOex3 (5’-GCTCANACTACRAATCCTA-3’) (Shih et al. 2022), and the newly designed primers LCOex2 (5’-ACACATCTTTAGAYTTGCAATCTAA-3’), LCOex3 (5’-ACACATCTTYARAYTTGCAATYTAA-3’), HCOs (5’-ACTTCDGGRTGDCCAAARAAYCA-3’) and HCOex0 (5’-GAYTCTTTTTTDCCDGAYTC-3’). The PCR conditions for the above primers were 50s 94 °C / 70s 45 °C / 60s 72 °C (denaturation/annealing/extension for 40 cycles), followed by 72 °C extension for 10 min. Sequences were obtained by automated sequencing (Applied Biosystems 3730) and were aligned with the aid of the MUSCLE function of MEGA (v. 11, Tamura et al. 2021), after verification with the complimentary strand. Sequences have been deposited into NCBI GenBank (accession numbers. shown in Table 1). Previously generated 16S sequences of *Candidiopotamon* from Taiwan (Shih et al. 2006) were also used for the construction of the phylogenetic tree.

For the combined 16S and COI dataset, the best-fitting models for sequence evolution of individual datasets were determined by PartitionFinder (v. 2.1.1, Lanfear et al. 2017), selected by the Bayesian information criterion (BIC). The best model, HKY+I+G, was subsequently applied for Bayesian inference (BI) analysis. The BI was performed with MrBayes (v. 3.2.6, Ronquist et al. 2012). The search was run with four chains for 10 million generations and four independent runs, with trees sampled every 1000 generations. The convergence of chains was determined by the average standard deviation of split frequency values below the recommended 0.01 (Ronquist et al. 2020), and the first 1100 trees were discarded as the burn in. The maximum likelihood (ML) analysis was conducted in RAxML (v. 7.2.6, Stamatakis 2006). The model GTR + G (i.e., GTRGAMMA) was used for all subsets with 100 runs to find the best ML tree by comparing likelihood scores. The robustness of the ML tree was evaluated by 1,000 bootstrap pseudoreplicates using the model GTRGAMMA. Basepair (bp) differences and pairwise estimates of Kimura 2-parameter (K2P) distances (Kimura 1980) for genetic diversities between specimens were calculated with MEGA.

## ﻿Results

### ﻿Genetic analyses

**Figure 2.**
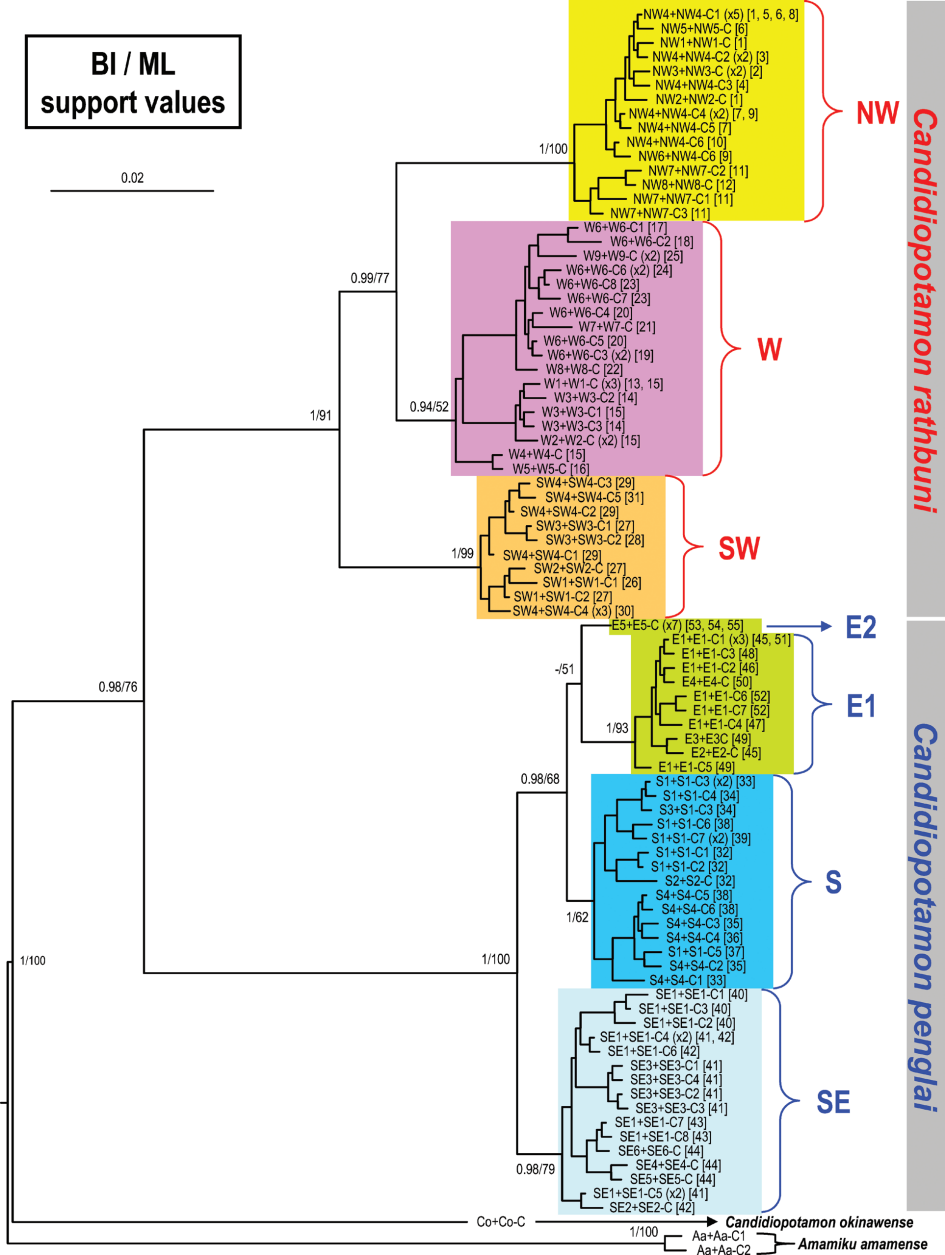
A Bayesian inference (BI) tree for *Candidiopotamonrathbuni* (De Man, 1914) and *Candidiopotamonpenglai* sp. nov., as well as outgroups, based on the combined 16S rDNA and cytochrome oxidase I. Probability values at the nodes represent support values for BI and maximum likelihood (ML). For haplotype names, see Table 1. The color of clades corresponds to the color of collection sites in Fig. 1, which are also shown as the numbers in parenthesis after the haplotype names.

A 553 bp segment of the 16S rDNA and a 658 bp segment of COI from 112 specimens of *C.rathbuni* and *C.penglai* sp. nov. were amplified and aligned. A total of 36 haplotypes of the 16S gene and 83 haplotypes of COI were found (Table 1). The phylogenetic tree constructed from BI analysis, with the respective confidence values from the ML analysis, is shown in Fig. 2. Only confidence values larger than 50% are shown. Based on Fig. 2 of the combined dataset, all *Candidiopotamon* from Taiwan are monophyletic, supported by BI and ML methods. Both the populations from western Taiwan (representing *C.rathbuni*) and eastern Taiwan (representing *C.penglai* sp. nov.) form two distinct major clades with high supports. In *C.rathbuni*, the SW clade was first isolated, and the other two clades (W and NW) form a larger clade. All three clades are highly supported, except for the ML method of the W clade. In *C.penglai* sp. nov., the SE clade diverged firstly, and the other three clades (S, E1, and E2) form a larger clade with E2 clade with a single haplotype (“E5+E5-C”) from northern Hualien. Three clades (SE, S, and E1) are highly supported, except for the ML method of S clade.

The pairwise nucleotide divergences and the total bp number differences of COI within and between clades and species are shown in Table 2. The genetic distances (and bp differences) within most clades are from 0 to 2.17% (0–14 bp), except the W clade with a larger value, 0–3.93% (0–25 bp). The genetic distances (bp differences) between clades are 3.92%–7.45% (25–46 bp) in *C.rathbuni*; and 1.7%–4.41% (11–28 bp) in *C.penglai* sp. nov. The intraspecific distances (bp differences) of *C.rathbuni* and *C.penglai* sp. nov. are 0–7.45% (0–46 bp) and 0–4.41% (0–28 bp), respectively. The interspecific distance (and bp difference) is 10.72%–13.65% (65–81 bp).

**Table 2.** Matrix of percentage pairwise nucleotide divergences with K2P distance and the number of bp differences based on COI within and between clades of *Candidiopotamon* species from Taiwan (see Table 1). In the right half, lower-left values are K2P distance and upper-right ones are bp differences. Values of the range are given in parentheses.

		Within clade		Between clades								
		Nucleotide divergence	bp difference	*C.rathbuni*			*C.penglai*				*C.rathbuni*	*C.penglai*
				NW	W	SW	S	SE	E1	E2		
***C.rathbuni***	NW	0.59 (0–1.54)	3.85 (0–10)		33.86 (25–43)	38.64 (35–44)	73.55 (69–80)	74.67 (72–81)	72.4 (69–78)	72.73 (71–76)		
	W	1.96 (0–3.93)	12.6 (0–25)	5.37 (3.92 –6.92)		37.17 (29–46)	70.93 (65–76)	72.1 (66–79)	69.75 (67–75)	70.63 (68–73)		
	SW	0.89 (0–1.85)	5.83 (0–12)	6.16 (5.54 –7.07)	5.95 (4.58 –7.45)		73.05 (66–79)	73.22 (68–78)	71.62 (69–75)	72.58 (70–74)		
***C.penglai***	S	1.36 (0–2.16)	8.81 (0–14)	12.25 (11.41 –13.48)	11.83 (10.72 –12.79)	12.19 (10.89 –13.34)		22.14 (18–28)	18.19 (15–21)	12.81 (11–15)		
	SE	1.12 (0–2.17)	7.25 (0–14)	12.45 (11.93 –13.65)	12.05 (10.9 –13.32)	12.23 (11.26 –13.13)	3.46 (2.79 –4.41)		22.18 (17–27)	21.83 (20–25)		
	E1	0.54 (0–1.07)	3.55 (0–7)	12.04 (11.41 –13.09)	11.61 (11.1 –12.6)	11.93 (11.44 –12.57)	2.83 (2.32 –3.28)	3.47 (2.64 –4.24)		13.83 (12–16)		
	E2	0	0	12.11 (11.79 –12.72)	11.79 (11.3 –12.24)	12.11 (11.62 –12.38)	2.06 (1.7 –3.28)	3.41 (3.12 –3.92)	2.15 (1.86 –2.49)			
***C.rathbuni***		4.18 (0–7.45)	26.34 (0–46)									72.34 (65–81)
***C.penglai***		2.53 (0–4.41)	16.26 (0–28)								12.06 (10.72 –13.65)	

### ﻿Taxonomy

**Family Potamidae Ortmann, 1896**

**Subfamily Potamiscinae Ortmann, 1896 (sensu Yeo and Ng 2004)**

#### ﻿Genus Candidiopotamon

Taxon classificationAnimaliaDecapodaPotamidae

Bott, 1967

79B1EE6B-64E8-5A69-AD46-7EF2FCACBDF3

Candidiopotamon Bott, 1967: 210; Bott 1970: 189; Minei 1974: 245; Dai 1999: 154; Shy and Yu 1999: 95; Ng et al. 2008: 161; Sasaki 2019: 11551; Shy et al. 2020: 1.

##### Type species.

Potamon (Potamon) rathbuni De Man, 1914.

##### Distribution.

Taiwan and the Ryukyu Islands (Okinawa, Kumejima, and Tokashiki islands) of Japan.

##### Remarks.

The genus *Candidiopotamon* was erected by Bott (1967) with the type species, Potamon (Potamon) rathbuni De Man, 1914, and the genus was named from the type locality of the type species, Candidius-See (= Sun Moon Lake, Rihyuetan) in Nantou Co. (= County), central-western Taiwan. *Candidiopotamon* was placed in the family Sinopotamidae Bott, 1970, by Bott (1970), but the Sinopotamidae was synonymized under the Potamidae by Yeo and Ng (2004), and this has been supported by genetic data (Shih et al. 2009). Following this study, there are now two species in Taiwan (*C.rathbuni* in the west and *C.penglai* sp. nov. in the east) and three from the Ryukyu Islands of Japan (*C.okinawense* in Okinawa Island, *C.kumejimense* in Kumejima Island, and *C.tokashikense* in Tokashiki Island). *Candidiopotamonguangdongense* Dai, 1999, claimed to be found in Guangdong, China, but this is a junior synonym of *C.rathbuni* and was based on incorrect locality labels (Shih and Ng 2011; Ng et al. 2017; Shy et al. 2020).

### ﻿Key to the species of *Candidiopotamon* Bott, 1967

**Table:** 

1	Distal part of G1 opening outwards, terminal segment relatively slender	**2**
–	Distal part of G1 opening distally, terminal segment relatively stout	**3**
2	Subterminal segment of G1 straight or curving inwards; distal inner edge of terminal segment of G1 with proportionally larger keel-like projection directed more distally	***C.penglai* sp. nov. (eastern Taiwan)**
–	Subterminal segment of G1 straight; distal inner part of terminal segment of G1 with proportionally smaller keel-like projection directed more laterally	***C.rathbuni* (De Man, 1914) (western Taiwan)**
3	Outer angle of frontal margin of carapace not touching first segment of endopod of antenna	***C.okinawense* Minei, 1973 (Okinawa Island, Japan)**
–	Outer angle of frontal margin of carapace touching first segment of endopod of antenna	**4**
4	Outer dorsal margin of second ambulatory propodus with 1 row of spines; subdistal carina of G1 directed dorsally	***C.tokashikense* Naruse, Segawa & Aotsuka, 2007 (Tokashiki Island, Japan)**
–	Outer dorsal margin of second ambulatory propodus without spines; subdistal carina of G1 directed inwards	***C.kumejimense* Minei, 1973 (Kumejima Island, Japan)**

#### ﻿ Candidiopotamon rathbuni

Taxon classificationAnimaliaDecapodaPotamidae

(De Man, 1914)

3CE29DCD-F9AC-5A3E-879E-7038516529B5

**Figure 3.**
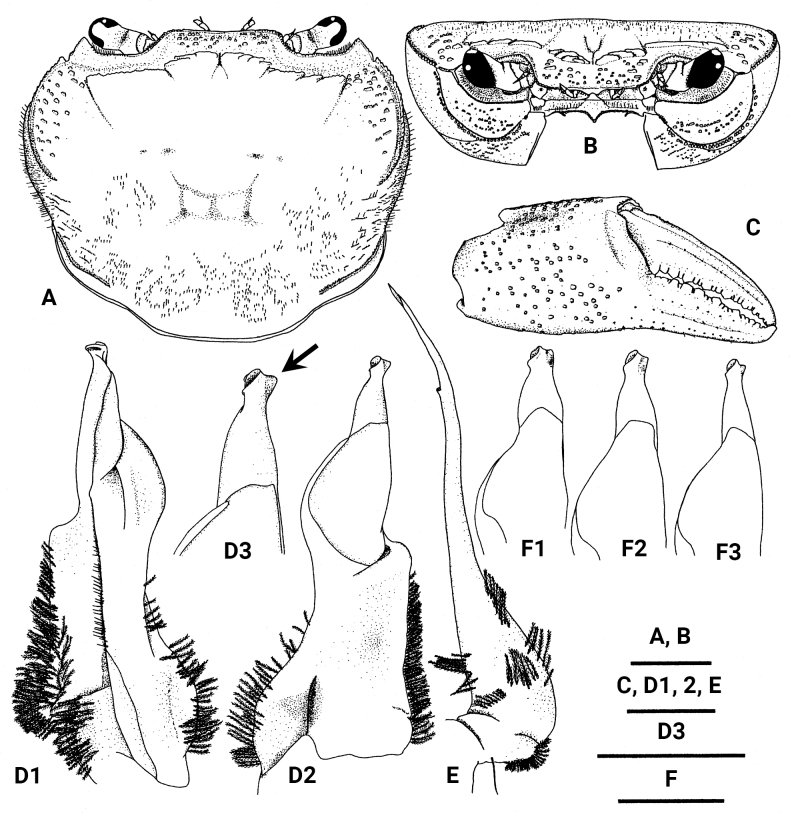
*Candidiopotamonrathbuni* (De Man, 1914): male (44.6 × 36.8 mm) from Chiayi, western Taiwan (RUMF-ZC-86) (**A**–**E**); male (35.4 × 31.2 mm) from Miaoli, northwestern Taiwan (NCHUZOOL 15194) (**F1**); male (36.7 × 31.1 mm) from Chiayi, western Taiwan (NCHUZOOL 15205) (**F2**); male (36.3 × 30.7 mm) from Taiwu, Pingtung, southwestern Taiwan (NCHUZOOL 15199) (**F3**). **A** carapace, dorsal view **B** carapace, frontal view **C** male major chela, outer view **D1** left G1, ventral view **D2** left G1, dorsal view **D3** left G1 terminal segment, dorsal view **E** left G2**F1–3** left G1s terminal segment, dorsal view. Scale bars: 10 mm (**A**–**C**); 2 mm (**D**–**F**). Arrow indicates the keel-like projection on the distal inner edge of the terminal segment.

**Figure 4.**
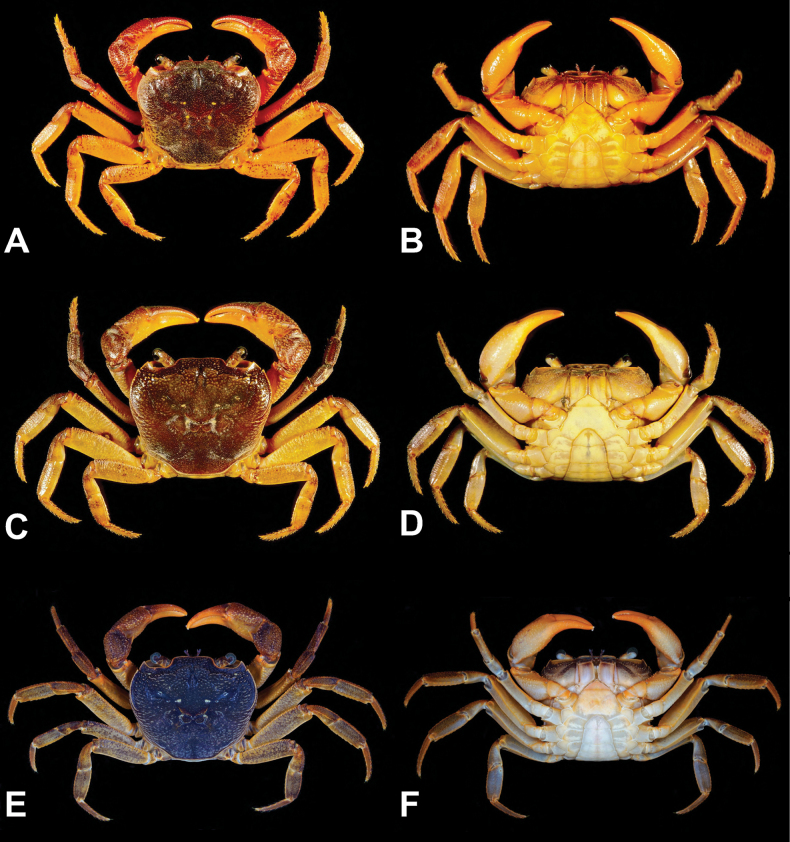
Color in life of *Candidiopotamonrathbuni* (De Man, 1914) (**A, B**) and *Candidiopotamonpenglai* sp. nov. (**C–F**). **A, C, E** overall dorsal view **B, D, F** overall ventral view **A, B**NCHUZOOL 15181 (male, 28.0 × 23.9 mm, Rihyuetan, Nantou, western Taiwan) **C, D**NCHUZOOL 15204 (male, 39.7 × 34.4 mm, Nanrenshan, Pingtung, southern Taiwan); NCHUZOOL 15224 (paratype male, 36.4 × 30.9 mm, Liyu Lake, Shoufeng, Hualien, eastern Taiwan).

**Figure 5.**
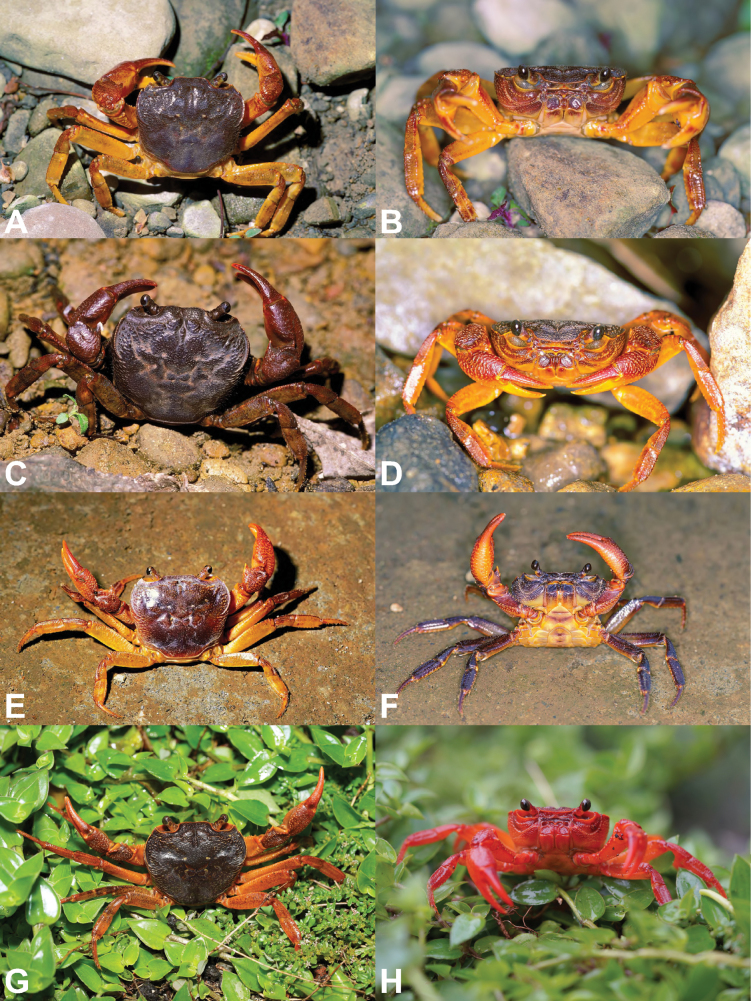
Color in life of *Candidiopotamonrathbuni* (De Man, 1914) (**A–D**) and *Candidiopotamonpenglai* sp. nov. (**E–H**). **A, B**NCHUZOOL 15194 (male, 35.4 × 31. 2 mm, Tai-an, Miaoli, northwestern Taiwan) **C**NCHUZOOL 15211 (male, 24.8 × 22.1 mm, Dongshih, Taichung, western Taiwan) **D**NCHUZOOL 15181 (male, 28.0 × 23.9 mm, Rihyuetan, Nantou, western Taiwan) **E, F**NCHUZOOL 15204 (2 males, 39.7 × 34.4 mm, 40.6 × 34.3 mm, Nanrenshan, Pingtung, southern Taiwan) **G**NCHUZOOL 15224 (paratype male, 36.4 × 30.9 mm, Liyu Lake, Shoufeng, Hualien, eastern Taiwan) **H**NCHUZOOL 15229 (male, 33.1 × 29.7 mm, Jili Lake, Guangfu, Hualien, eastern Taiwan).

Figs 3 , 4A, B , 5A–D

Potamon (Potamon) rathbuni De Man, 1914: 128, pl. 3(4–4d) [type locality: Sun Moon [Rihyuetan], Nantou, Taiwan]; Parisi 1916: 153; Oshima 1921: 123; Balss 1922: 134; Maki and Tsuchiya 1923: 153, pl. 19(2); Balss 1937: 162, fig. 21; Sakai 1939: 580, pl. 128(1); Sakai 1940: 57; Lin 1949: 25; Chiu 1962a: 425.
Thelphusa rubra Nakagawai Nakagawa, 1915a: 1036 (nomen nudum); 1915b: 322.
Potamon (Geothelphusa) obtusipes – Terao 1915: 503, 1 unnumbered fig.; Nakagawa 1917: 303, fig. 21 (non Geothelphusaobtusipes Stimpson, 1858).
Potamon obtusipes – Nakagawa 1916: 137, pl. 2(1) (non Geothelphusaobtusipes Stimpson, 1858).
Potamon rathbuni – Koba 1936a: 166, fig. 1; Koba 1936b: 202, text-fig.; Horikawa 1940: 27; Iwata 1940: 2; Chiu 1962b: 58; Chiu 1964: 67 (part); Pretzmann 1963: 365, fig. 15.
Candidiopotamon rathbuni – Bott 1967: 210, fig. 10; Bott 1970: 189, pls 40(74), 55(75); Minei 1974: 246, figs 7–9 (part); Sakai 1976: 564, text-fig. 307; Dai et al. 1984: 115, fig. 69; Hwang and Mizue 1985: 10, fig. 7, pl. IIA (part); Shy et al. 1996: 239, fig. 17; CH Jeng et al. 1998: 70, 2 unnumbered figs; Dai 1999: 154, fig. 80, pl. 9(7) (part); Shy and Yu 1999: 94, 3 unnumbered figs, “fig. 31” on p. 106 (part); Lee and Tung 2000: 70; Ng 2000: 249 (part); Jeng et al. 2010: 80, 7 unnumbered figs; Ng et al. 2017: 76; Shy et al. 2020: 2, figs 11A, 13, 14, 16, 17 (part).
Candidiopotamon sp. – Jeng et al. 1994: 56; Jeng et al. 1996: 51; Jeng et al. 1997: 62; MS Jeng et al. 1998: 60.
Candidiopotamon guangdongense Dai, 1999: 156, fig. 81, pl. 19(8).
Candidiopotamon rathbunae – Liu and Li 2000: 90; Ng 2000: 249 (part); Chen et al. 2001: 43, 2 unnumbered figs on p. 43, 46 (part); Lee 2001: 145, 2 unnumbered figs (part); Ng et al. 2001: 49 (part); Chen et al. 2003: 29, 5 unnumbered figs (part); Naruse et al. 2004: 7, fig. 2Bd, Bv.; Shih et al. 2006: 983 (part); Ng et al. 2008: 161 (part); Shih et al. 2009: 706; Shy and Lee 2009: 207, 10 unnumbered figs (part); Chiu et al. 2015: 202, 227, 1 unnumbered fig. on p. 202 (part); Ho 2015: 141, 2 unnumbered figs (part); Liu and Hartnoll 2017: 227; Sasaki 2019: 11552 (part).

##### Material examined.

Taiwan — 3 males, CW 25.6 × CL 22.6–28.6 × 25.6 mm, 1 female, 38.0 × 32.8 mm, NCHUZOOL 15191, Sinsian, Wulai, New Taipei City, coll. C. A. Chen, 20 Aug. 2001; 1 male, 40.0 × 35.8 mm, NCHUZOOL 15189, Sinsian, Wulai, New Taipei City, coll. H.-T. Shih, 29 Apr. 1994; 1 male, 30.2 × 27.4 mm, 1 female, 35.1 × 30.7 mm, NCHUZOOL 12915, Jianshih, Hsinchu Co., coll. H.-T. Shih, 25 May 2001; 1 female, 40.0 × 34.0 mm, NCHUZOOL 15184, Hengshan, Hsinchu Co., coll. H.-C. Liu, 24 Sep. 1995; 2 males, 33.4 × 29.1–32.8 × 29.0 mm, 1 female, 33.9 × 29.0 mm, RUMF-ZC-53, Cingcyuan, Wufeng, Hsinchu Co., coll. C.A. Chen, 28 Aug. 2001; 1 male, 30.9 × 27.0 mm, NCHUZOOL 15192, Shueiweiping, Dahu, Miaoli, coll. H.-D. Zhu, 7 Mar. 2001; 3 males, 25.7 × 22.6–35.4 × 31.2 mm, 1 female, 26.7 × 23.9 mm, NCHUZOOL 15194, Baguali, Tai-an, Miaoli, coll. H.-T. Shih, 25 Jan. 2002; 1 male, 34.5 × 30.6 mm, NCHUZOOL 15211, 2 males, 19.1 × 16.5, 19.8 × 17.6 mm, 5 females, 17.9 × 15.9–31.0 × 26.9 mm, NCHUZOOL 12918, 1 male, 31.6 × 27.5 mm, 1 female, 27.9 × 23.8 mm, RUMF-ZC-54, Sihjiaolin, Dongshih, Taichung, coll. H.-T. Shih, 28 Dec. 2001; 1 male, 23.3 × 20.3 mm, NCHUZOOL 12944, Caohu, Taichung, coll. H.-C. Liu, 23 Apr. 1993; 1 male, 30.4 × 26.1 mm, NCHUZOOL 12924, Taiping, Taichung, coll. students of Tunghai Univ., 8 Jun. 2001; 2 males, 26.4 × 23.2, 27.7 × 24.2 mm, NCHUZOOL 15214, Lianhuachih, Wucheng, Yuchih, Nantou, coll. H.-T. Shih, 22 Jun. 2001; 1 male, 28.0 × 23.9, 1 female, 29.7 × 25.4 mm, NCHUZOOL 15181, Rihyuetan, Nantou (type locality), coll. H.-T. Shih and H.-T. Hung, 4 Mar. 2003; 1 male, 38.6 × 32.4 mm, NCHUZOOL 15187, Sinshan, Shueili, Nantou, coll. H.-C. Liu, 8 Aug. 1995; 1 male, 36.3 × 30.9, 1 female, 24.2 × 21.0 mm, NCHUZOOL 15185, Jiji, Shueili, Nantou, coll. H.-C. Liu, 8 Aug. 1995; 3 males, 32.3 × 27.6–36.7 × 31.1 mm, 2 females, 26.4 × 22.5, 33.0 × 28.7 mm, NCHUZOOL 15205, Chukou, Fanlu, Chiayi Co., coll. H.-C. Liu, 21–22 Mar. 1996; 1 male, 44.6 × 36.8 mm, 1 female, 40.9 × 34.4 mm, RUMF-ZC-86, Guanyin Waterfall, Chiayi Co., coll. J.-Y. Shy, 6 Dec. 1990; 1 male, 35.2 × 29.3 mm, NCHUZOOL 12946, Alishan, Chiayi Co., coll. C.-H. Wang, 26 May 2002; 1 male, 21.2 × 19.1 mm, NCHUZOOL 15182, Dakeng, Dongyuan, Dongshan, Tainan, coll. H.-T. Shih, 20 Oct. 2000; 5 males, 19.7 × 16.8–29.6 × 25.6 mm, 1 female, 24.9 × 20.9 mm, NCHUZOOL 15186, Gaoyuan, Dongshan, Tainan, coll. H.-C. Liu, 16 Aug. 1997; 1 male, 26.6 × 22.6 mm, NCHUZOOL 15196, Gueidan, Nansi, Tainan, coll. H.-T. Shih, 20 Oct. 2000; 1 male, 28.8 × 24.9 mm, NCHUZOOL 15198, Mincyuan, Sanmin, Kaohsiung, coll. C.-B. Wu, 17 Aug. 2000; 1 male, 30.6 × 26.0 mm, 1 female, 25.0 × 21.8 mm, NCHUZOOL 15195, Baolai, Liouguei, Kaohsiung, coll. H.-T. Shih, 14 Dec. 2001; 1 male, 31.5 × 26.6 mm, NCHUZOOL 12922, Chuyunshan, Taoyuan, Kaohsiung, coll. C.-S. Chen, 6 May 2000; 1 male, 34.3 × 29.0 mm, NCHUZOOL 15188, Guanglin, Meinong, Kaohsiung, coll. C.-S. Chen, 15 Dec. 2000; 3 females, 33.7 × 29.7–40.4 × 35.0 mm, NCHUZOOL 15183, Guanglin, Meinong, Kaohsiung, coll. H.-C. Liu, 4 Aug. 1995; 1 male, 37.3 × 32.3 mm, 1 female, 26.7 × 23.2 mm, NCHUZOOL 15190, Jiasian, Kaohsiung, coll. H.-C. Liu, 5 Aug. 1995; 3 males, 30.5 × 26.2–38.8 × 34.0 mm, 1 female, 27.8 × 23.8 mm, NCHUZOOL 15197, Tianliao, Kaohsiung, coll. H.-T. Shih, 16 Nov. 2001; 1 male, 24.2 × 20.5 mm, 1 female, 26.2 × 22.3 mm, NCHUZOOL 15202, Shandimen, Pingtung, coll. H.-C. Liu, 16 Aug. 1997; 1 male, 34.4 × 29.4 mm, NCHUZOOL 15201, Haocha, Pingtung, coll. H.-T. Shih, 31 May 2000; 2 males, 36.3 × 30.7, 37.7 × 32.3 mm, 1 female, 32.9 × 27.3 mm, NCHUZOOL 15199, Taiwu, Pingtung, coll. C.-C. Huang and C.-K. Yang, 29 Jul. 1999; 1 male, 34.5 × 29.0 mm, 1 female, 37.4 × 30.9 mm, RUMF-ZC-55, Taiwu, Pingtung, coll. H.-T. Shih, 12 Nov. 2000; 2 males, 33.4 × 28.3, 37.5 × 31.7 mm, NCHUZOOL 15203, Wutan, Taiwu, Pingtung, coll. H.-C. Liu, 18 Jul. 1997; 3 females, 13.2 × 11.3–29.5 × 24.9 mm, NCHUZOOL 12925, Laiyi, Pingtung, coll. H.-C. Liu, 16 Jul. 1996.

##### Diagnosis.

Carapace subtrapezoidal, CW 1.10–1.21× CL (mean 1.16, *n* = 5), dorsal surface almost flat, relatively rough, epigastric and postfrontal cristae distinct, both cristae represented by 1–3 oblique lines of granules, lateral end of postfrontal cristae far apart from epibranchial tooth; external orbital and epibranchial teeth distinct, sharp, directed anteriorly. G1 terminal segment proportionally longer, more slender, distal opening directed laterally, distal inner edge with keel-like projection in dorsal view, projection proportionally small, directed more laterally; G1 subterminal segment straight.

##### Description.

Carapace (Figs 3A, B, 4A) subtrapezoidal in dorsal view, CW 1.10–1.21× CL (mean 1.16, *n* = 5), dorsal surface almost flat, relatively rough, somewhat pilose. Epigastric cristae distinct; postorbital crista, represented by 1–3 oblique lines of granules, not reaching epibranchial tooth. External orbital angle acute, directed anteriorly; ridge between external orbital angle and distinct epibranchial tooth cristate and granulated; anterolateral margin distinctly cristate, lined with large, elongated granules.

Antenna (Fig. 3B) short, reaching ~ 1/2 length of basal segment of antenna when antenna folded backwards.

Eye (Fig. 3B) developed; maximal width of cornea wider than base of peduncle in frontal view.

Chelae of large males unequal, females or young specimens with subequal chelae; palm of major chela (Fig. 3C) ~ 2/3×as high as minor chela, outer surface with scattered granules; cutting edges of both fingers with regularly arranged large and small teeth, gape almost absent when fingers closed.

Ambulatory legs moderately long; propodus cross-section oblong, each of 2 inner and 1 ventral outer longitudinal margins with 1 spine-row.

Male pleonal somites elongate trapezoidal, sixth pleonal somite length ~ 1/2 of its width; telson obtusely triangular, length ~ 1/2 of its width, distal end reaching imaginary line joining lower hinges of cheliped coxae. Female pleon of adult wide, covering ventral surface of thoracic sternum except for part of thoracic sternites 1 and 2.

G1 (Fig. 3D, F) straight in dorsal view when dorso-proximal margin of subterminal segment set parallel to observer’s eyes. Ventral side of subterminal segment with median seam extending to subproximal part of terminal segment. Synovial membrane long, wide, occupying distal ~ 2/5 of subterminal segment in dorsal view. Terminal segment bottle-necked, relative width gradually narrowed from northwestern (Fig. 3F1) to southwestern populations (Fig. 3F3), but no clear gap between them (Fig. 3F); distal opening directed outwards, with relatively small, laterally directed keel-like projection on opposite side of opening. G2 slightly shorter than G1; G2 flagellum ~ 1/4 length of G2 total length.

Vulvae close to each other, obliquely oblong, occupying proximal two-thirds of thoracic sternite 6.

##### Coloration

**(Figs 4A, B, 5A–D).** Most large specimens are dark red, but sometimes dark purple or orange individuals can be found. Small individuals are generally dark brown with black spots.

##### Distribution

**(Fig. 1).***Candidiopotamonrathbuni* is distributed along the western side of the Central Range, i.e., New Taipei City, Taoyuan, Hsinchu Co., Miaoli, Taichung, Changhua, Yunlin, Chiayi Co., Tainan, Kaohsiung, and the northwestern part of Pingtung. The type locality of *C.rathbuni* is Rihyuetan (= Sun Moon Lake, or the Candidius Sea), Nantou, west of the Central Range of Taiwan.

##### Habitat.

Most large specimens were found under large stones within streams. Sometimes adults move outside the water at night.

##### Remarks.

Based on one male (31.4 × 26 mm) and one female (37 × 31.3 mm) specimens, De Man (1914) described the morphology of *Candidiopotamonrathbuni* in great detail except for the gonopods. The lack of a description of G1 makes it difficult to ascertain which species is the real *C.rathbuni*. The topotypic specimens of *C.rathbuni* (NCHUZOOL 15187, 15185, 15214) possess the laterally directed subdistal keel and straight subterminal segment of G1. Furthermore, the type locality of *C.rathbuni*, Sun Moon Lake (= Rihyuetan), is located in west-central Taiwan (i.e., west of the Central Range, < 1000 m, a.s.l), which suggests that the species distributed in western Taiwan keeps the name *C.rathbuni*, and that of eastern Taiwan is herewith described as a new species.

In his papers on the life history of *Paragonimuswestermani*, Nakagawa (1915a, 1915b) used “*Thelphusarubra Nakagawai*” for the specimens collected by himself from Hsinchu (including Miaoli), northwestern Taiwan as the second intermediate host of the species, without further information on the specimens he observed. We have examined the specimens from Hsinchu (NCHUZOOL 12915, 15184, RUMF-ZC-53) and Miaoli (NCHUZOOL 15192, 15194), which belong to *C.rathbuni* from western Taiwan in morphology and genetics. Regarding the brick-red coloration from the name “rubra”, the coloration of *C.rathbuni* is variable from dark purple and dark red to orange (see “Coloration” of this species).

Subsequently, Terao (1915) referred Nakagawa’s specimens of “*Thelphusarubra Nakagawai*” to Potamon (Geothelphusa) obtusipes (Stimpson, 1858). However, the real *P.obtusipes* is only distributed in Amami Islands, the Ryukyus and does not show the red coloration (Shih 2008: figs 51, 52). Terao’s (1915: 508) drawing of P. (G.) obtusipes clearly showed that his specimen represented a species of *Candidiopotamon*, and Ng et al. (2001) also doubted that “*Thelphusarubra Nakagawai*” was actually *C.rathbuni*. In addition to the above history, Nakagawa stated that “As a matter of convenience, the crab, which has been described as *Thelphusa* sp., is provisory treated here (as *Thelphusarubra Nakagawai*) temporarily”. Because Nakagawa collected the specimens from Hsinchu and Miaoli, northwestern Taiwan where *C.rathbuni* is distributed, “*Thelphusarubra Nakagawai*” sensu Nakagawa (1915a, 1915b) and Potamon (Geothelphusa) obtusipes sensu Terao (1915) are considered here as junior synonyms of *C.rathbuni*.

#### ﻿ Candidiopotamon penglai sp. nov.

Taxon classificationAnimaliaDecapodaPotamidae

FB5DB50C-9681-5662-AFBA-ECC7F366EA46

https://zoobank.org/3AC24730-7610-4C20-8F55-963FE8C56D6A

**Figure 6.**
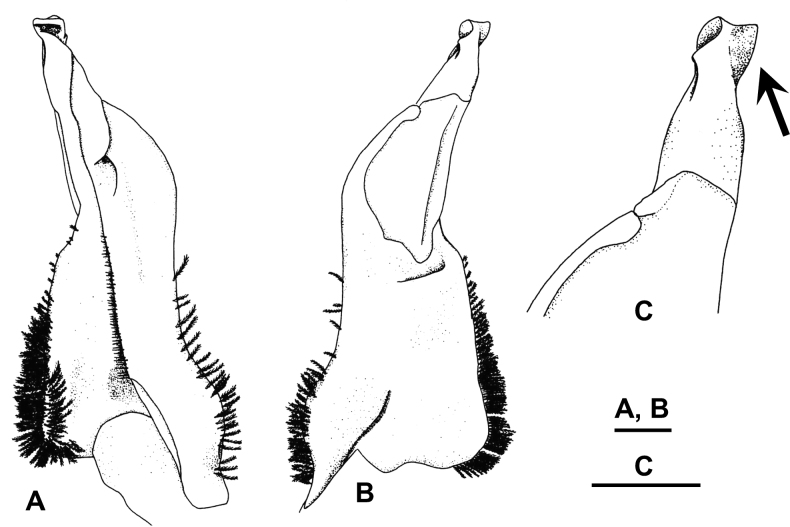
*Candidiopotamonpenglai* sp. nov., holotype male (38.4 × 33.4 mm) from Ji-an, Hualien (NCHUZOOL 12951), G1 in bent form **A** left G1, ventral view **B** left G1, dorsal view **C** left G1 terminal segment, dorsal view. Scale bars: 1 mm. Arrow indicates the keel-like projection on the distal inner edge of the terminal segment.

**Figure 7.**
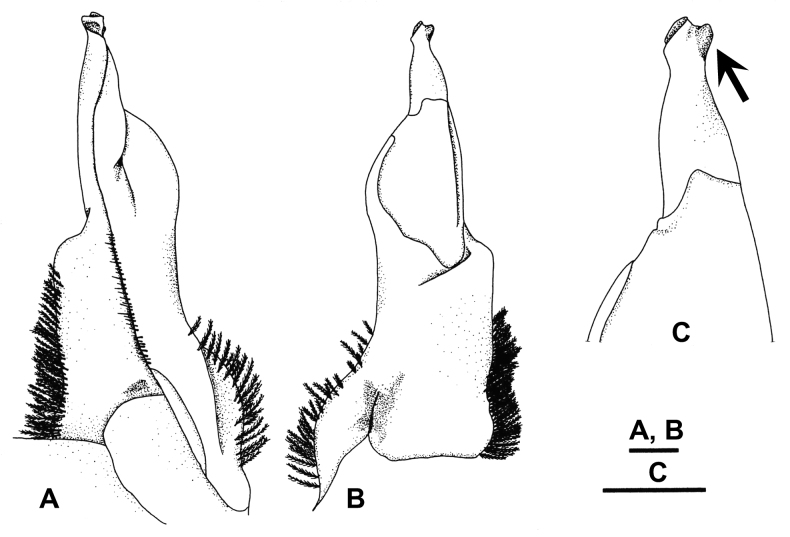
*Candidiopotamonpenglai* sp. nov., male (42.9 × 36.2 mm) from Shihzih, Pingtung (NCHUZOOL 15207), G1 in straight form **A** left G1, ventral view **B** left G1, dorsal view **C** left G1 terminal joint, dorsal view. Scale bars: 1 mm. Arrow indicates the keel-like projection on the distal inner edge of the terminal segment.

Figs 4C–F , 5E–H , 6 , 7

Potamon rathbuni – Chiu 1964: 67 (part).
Candidiopotamon rathbuni – Minei 1974: 246, figs 7–9 (part); CH Wang 1984: 41; Yu et al. 1996: 9, 29, fig. 10; MS Jeng 1998: 86, 1 unnumbered fig.; Shy and Yu 1999: 94, 3 unnumbered figs, “fig. 31” on p. 106 (part); Ng 2000: 249 (part); Ng et al. 2017: 76; Shy et al. 2020: 2, figs 11A, 13, 14, 16, 17 (part); Li and Chiu 2019a: 103, 3 unnumbered figs; Li and Chiu 2019b: 2 unnumbered figs on p. 56; Shy et al. 2020: 2, figs 2E, 14E (part); SW Wang et al. 2021: 32, pl. 3E.
Candidiopotamon rathbunae – Ng 2000: 249 (part); Chen et al. 2001: 43, 2 unnumbered figs on p. 43, 46 (part); Lee 2001: 145, 2 unnumbered figs (part); Ng et al. 2001: 49 (part); Chen et al. 2003: 29, 5 unnumbered figs (part); Shih et al. 2006: 983 (part); Ng et al. 2008: 161 (part); Shy and Lee 2009: 207, 10 unnumbered figs (part); Li and Chiu 2013: 70, 3 unnumbered figs; Chiu et al. 2015: 202, 227, 1 unnumbered fig. on p. 202 (part); Ho 2015: 141, 2 unnumbered figs (part); Sasaki 2019: 11552 (part).

##### Type material.

Taiwan — ***Holotype***: male, 38.4 × 33.4 mm, NCHUZOOL 12951, Ji-an, Hualien, coll. H.-C. Liu, 4 May 2000. — ***Paratypes***: 1 male, 35.6 × 30.5 mm, RUMF-ZC-8164, 1 female, 18.0 × 15.8 mm, NCHUZOOL 12932, collection data same as for holotype; 1 female, 34.3 × 29.6 mm, RUMF-ZC-8165, Shueiyuandi, Shoufeng, Hualien, coll. N.-H. Jang-Liaw, 5 Mar. 2002; 1 male, 23.4 × 20.0 .5 mm, NCHUZOOL 12952, Liyu Lake, Shoufeng, Hualien, coll. H.-T. Shih, 26 Jan. 2007; 1 male, 27.5 × 24.2 mm, NCHUZOOL 15213, Liyu Lake, Shoufeng, Hualien, coll. H.-T. Shih, 28 Jan. 2007; 1 male, 36.4 × 30.9 mm, NCHUZOOL 15224, 1 male, 27.0 × 23.8, NCHUZOOL 15226, 1 female, 36.5 × 31.4, NCHUZOOL 15225, Liyu Lake, Shoufeng, Hualien, coll. HTS’s lab students, 13 Apr. 2023; 1 male, 28.3 × 24.8 mm, 1 female, 35.2 × 31.6 mm, ZRC 2023.0309, Liyu Lake, Shoufeng, Hualien, coll. HTS’s lab students, 13 Apr. 2023.

##### Other material.

Taiwan — 1 male, 23.9 × 20.1 mm, NCHUZOOL 15227, 1 male, 29.3 × 27.6 mm, NCHUZOOL 15228, 1 male, 33.1 × 29.7 mm, NCHUZOOL 15229, Jili Lake, Guangfu, Hualien, coll. HTS’s lab students, 12 Apr. 2023; 1 female, 37.7 × 33.6 mm, NCHUZOOL 15230, Luoshan, Fuli, Hualien, coll. HTS’s lab students, 11 Apr. 2023; 2 males, 35.9 × 30.6, 37.0 × 31.9 mm, 2 females, 27.6 × 23.5, 36.7 × 31.9 mm, RUMF-ZC-56, Jingpu, Hualien, coll. M.-Y. Liu, 18 Jun. 2001; 2 males, 33.0 × 28.9, 30.4 × 26.5 mm, NSMT-Cr 15170, Jingpu, Hualien, coll. M.-Y. Liu, Jan. 2001; 1 male, 31.7 × 26.7 mm, NCHUZOOL 15221, Chenggong, Taitung, coll. N.-H. Jang-Liaw, 6 Mar. 2002; 3 males, 28.2 × 23.3–34.0 × 28.8 mm, 2 females, 16.4 × 13.6, 38.3 × 31.0 mm, NCHUZOOL 12931, Taimali, Taitung, coll. H.-C. Liu, 22 Aug. 1997; 4 males, 28.6 × 24.8–33.7 × 29.1 mm, 1 female, 32.5 × 27.0 mm, NCHUZOOL 15206, Sansiantai, Chenggong, Taitung, coll. H.-C. Liu and H.-L. Hsu, 5 Sep. 1997; 2 males, 31.4 × 25.9, 27.0 × 23.3 mm, NCHUZOOL 12933, Lijia, Beinan, Taitung, 1250 m a.s.l, coll. S.-P. Wu, 22 Aug. 2001; 1 male, 28.4 × 24.6, NCHUZOOL 12938, 32.9 × 28.7 mm, ZRC 2003.0036, Luanshan, Yanping, Taitung, coll. H.-T. Shih, 10 Jan. 2001; 4 males, 29.0 × 24.6–35.6 × 29.3 mm, 1 female, 21.9 × 18.4 mm, NCHUZOOL 15218, Taiban, Daren, Taitung, coll. H.-C. Liu, 22 Apr. 1995; 1 female, 44.7 × 35.9 mm, NCHUZOOL 15210, Dawu, Taitung, coll. H.-C. Liu and C.-H. Wang, 22 Aug. 1997; 1 male, 28.9 × 24.1 mm, 1 female, 29.8 × 25.0 mm, NCHUZOOL 12943, Dawu, Taitung, coll. H.-T. Shih, 9 Jan. 2001; 2 males, 40.1 × 33.6, 42.9 × 36.2 mm, 1 female, 38.4 × 31.5 mm, NCHUZOOL 15207, Danlu, Shihzih, Pingtung, coll. H.-T. Shih, 12 Mar. 1999; 1 male, 28.0 × 23.3 mm, NCHUZOOL 15208, Wangsha, Hengchun, Pingtung, coll. H.-T. Shih, 5 Mar. 2000; 1 male, 31.0 × 25.2 mm, RUMF-ZC-57, Wangsha, Hengchun, Pingtung, coll. H.-T. Shih, 31 Mar. 1999; 2 males, 41.7 × 35.0, 41.9 × 35.0 mm, 1 female, 35.7 × 29.8 mm, NCHUZOOL 15217, Nanrenshan, Manjhou, Pingtung, coll. H.-T. Shih, 19 Oct. 1996; 3 males, 37.0 × 30.8–40.6 × 34.7 mm, NCHUZOOL 15204, Nanrenshan, Manjhou, Pingtung, coll. R.-H. Lee, 8 Feb. 2003; 1 male, 44.3 × 37.0 mm, ZRC 2003.0037, Nanrenshan, Manjhou, Pingtung, coll. H.-T. Shih, 24 Feb. 1999; 1 male, 43.8 × 36.7 mm, RUMF-ZC-58, Kenting, Hengchun, Pingtung, coll. H.-T. Shih, 6 Jan. 2000; 1 male, 30.6 × 26.2 mm, 1 female, 35.2 × 29.3 mm, NSMT-Cr 15171, Sihchong R., Mudan, Pingtung, coll. H.-T. Shih, 10 Mar. 1999 ; 1 male, 38.7 × 32.4 mm, 3 females, 38.3 × 33.5–43.6 × 35.9 mm, NCHUZOOL 15209, Nanshih, Shihzih, Pingtung, coll. H.-C. Liu, 22 Jul. 1997.

##### Diagnosis.

Carapace subtrapezoidal, CW 1.14–1.24× CL (mean 1.18, *n* = 45), dorsal surface almost flat, relatively rough, epigastric and postfrontal cristae distinct, both cristae represented by 1–3 oblique lines of granules, lateral end of postfrontal cristae far apart from epibranchial tooth; external orbital and epibranchial teeth distinct, sharp, directed anteriorly. G1 terminal segment proportionally longer and slender, distal opening directed laterally, distal inner edge with keel-like projection in dorsal view, projection proportionally large, directed more distally; G1 subterminal segment curving inwards (in eastern to southeastern Taiwan populations) or straight (in southern Taiwan populations).

##### Etymology.

The new species is named after Penglai, the ancient name of Taiwan. It also refers to the fact that the island of Taiwan was formed by the Penglai Orogeny (ca 5 million years ago) (Teng 1990; Liu et al. 2000), and the ancestral *Candidiopotamon* most likely colonized Taiwan at that time (Shih et al. 2006). The species name is used as a noun in apposition.

##### Coloration

**(Figs 4C–F, 5E–H).** Same as *C.rathbuni*, but young individuals tend to be reddish (e.g. Fig. 5H).

##### Distribution

**(Fig. 1).***Candidiopotamonpenglai* sp. nov. is distributed along the eastern side of the Central Range of Taiwan (except for the northeastern area), i.e., Hualien and Taitung in eastern Taiwan, as well as Hengchun Peninsula (the southern part of Pingtung and the southern tip of Taitung) in southern Taiwan. The type locality of *C.penglai* sp. nov. is Ji-an, Hualien (Fig. 1: no. 55).

##### Habitat.

Same as *C.rathbuni*.

##### Remarks.

A detailed morphological comparison reveals that specimens of *C.rathbuni* from western Taiwan and *C.penglai* sp. nov. from eastern Taiwan show differences in G1 structure. *Candidiopotamonrathbuni* has a smaller and more laterally directed keel-like projection on the distal inner part of the terminal segment (Fig. 3D3) [vs larger and more distally directed structure in *C.penglai* (Figs 5C, 6C)]. Furthermore, *C.rathbuni* show almost straight subterminal segment of G1 (Fig. 3D2), whereas those of *C.penglai* are varied either straight (S clade, Fig. 7) or bent (E1, E2 and SE clades, Fig. 6) (Figs 1, 5B, 6B; see Discussion).

This new species includes clades from eastern Taiwan (E1, E2, SE and S, see Fig. 1) that are genetically distinct from *C.rathbuni* from western Taiwan (NW, W and SW) (Table 2, Fig. 2), with a reliable character of G1 also separating the two species (see above). Within *C.penglai* sp. nov., there are two forms of the curvature in G1, with the bent form found in the E1, E2 and SE clades, and the straight form in the S clade (Fig. 1; see Discussion).

## ﻿Discussion

The phylogenetic pattern of *Candidiopotamon* in Taiwan inferred by the combined 16S and COI (Fig. 2) agrees with the phylogeny exclusively based on 16S (Shih et al. 2006). But with the inclusion of the COI marker and specimens from more sites in NW and E2 clades (Fig. 1), the tree in this study obtains higher nodal supports, and more distinct clades can be observed (Fig. 2). The separation of the eastern and western clades was proposed to be caused by the isolating effect of the Taiwan Central Range during 5.7 ± 1.1 million years ago (Shih et al. 2006).

The genetic distance between these two major clades from eastern and western Taiwan is ≥ 10.72% (K2P distance) (Table 2), which is much higher than most recognized species within the Potamidae, e.g., the minimum interspecific distances are between 1.31%–3.17% in *Geothelphusa* spp.; 6.7% in *Johora* spp.; 9.97% in *Lacunipotamon*; 2.17% in *Nanhaipotamon* spp.; 1.8% in *Longpotamonyangtsekiense* complex; and 6.22%–7.59% in *Tiwaripotamon* (see Chu et al. 2015; Do et al. 2016; Huang et al. 2020; Shy et al. 2021). Representatives of the two major clades are very similar in general morphology but can still be usually distinguished by the structure of the G1 (Fig. 1; see Remarks under *C.rathbuni*). As a result, we establish a new species, *Candidiopotamonpenglai* sp. nov., for the eastern major clade. *Candidiopotamonpenglai* sp. nov. can be considered a pseudocryptic species (i.e., minor morphological difference, and only receiving species status after other methods have been deployed to strengthen the case; as introduced by Ragionieri et al. 2012 and defined by Chu et al. 2015). Similar cases have been reported in several other brachyuran crabs (e.g., Ragionieri et al. 2012; Shih et al. 2013, 2018; Ng and Shih 2014, 2015, 2023; Lai et al. 2017; Fratini et al. 2019; Prema et al. 2022).

The maximum intraspecific distances within the Taiwanese species of *Candidiopotamon* are large, with 7.45% in *C.rathbuni* and 4.41% in *C.penglai* sp. nov. (Table 2). The large maximum intraspecific distances in some species of freshwater crabs have been proposed to be caused by the wide distribution with different degrees of geographical barriers, e.g., 3.33% in *Geothelphusatakuan* Shy, Ng & Yu, 1994 in northwestern Taiwan (Shy et al. 2021) and 5.25% in *Tiwaripotamonpluviosum* Do, Shih & Huang, 2016 in the boundary region between Vietnam and China (Do et al. 2016).

In *C.rathbuni*, three clades are recognizable, NW, W and SW (Fig. 2), with NW and W being closely related. In the present study, we found morphological variations in the G1 of *C.rathbuni*: the width of the subterminal segment gradually becomes stouter from northwestern to southwestern clades (Fig. 1). The morphological differences, however, are too subtle to recognize each clade consistently. In *C.penglai* sp. nov., both S and SE clades are monophyletic, but two clades are found in the eastern region between Beinan R. and Liwu R. (Figs 1, 2). The morphological study shows that the E1, E2 and SE clades have bent G1, but the G1 in the S clade is straighter (Fig. 1). The curvature or sinuosity of the G1 has been often used as an important character in taxonomic work on freshwater crabs. It is known that the shape of G1 can be variable. Brandis et al. (1999) showed that the terminal segment of G1 can be passively bent in its flexible zone, and that the shape of the flexible zone is responsible for the direction of deflection of the terminal segment. In the case of the E1, E2 and SE clades of *C.penglai* sp. nov., since the subterminal segment of the G1 is bent sideward direction (mesially) constantly from the synovial membrane, it is difficult to attribute the curvature to the passive bent of the flexible zone.

In a paper dealing with the freshwater decapod fauna of Yunlin, Chiayi, and Tainan counties of western Taiwan, Shy et al. (1996) diagnosed G1 of *C.rathbuni* as “curving inwards”, and the drawing of G1 showed a bent shape as well (Shy et al. 1996: fig. 17c, d). This description and figure were followed by Shy and Yu (1999: 97, “fig. 31” on p. 106) and Shy et al. (2020: 5, fig. 16). This specimen used, NTOU F10129 (Department of Environmental Biology and Fisheries Science, National Taiwan Ocean University, Keelung, Taiwan), was collected from Meishan, Chiayi Co., but the G1 was dried before (Jhy-Yun Shy personal communication), which may explain, why the G1 is more sinuous, unlike those from western clades.

## ﻿Conclusions

In this study, a new pseudocryptic species, *Candidiopotamonpenglai* sp. nov., from eastern Taiwan is established based on minor morphological differences of the G1 and pronounced differences in mitochondrial 16S and COI sequences. Morphological variation of the G1 was found in both *C.rathbuni* and *C.penglai* sp. nov. In *C.rathbuni*, a tendency from robust G1 in the northwestern population to slender G1 in southwestern populations was observed. In *C.penglai* sp. nov., northern and southern populations show the bent and straight form in the subterminal segment of G1, respectively.

## Supplementary Material

XML Treatment for Candidiopotamon

XML Treatment for Candidiopotamon rathbuni

XML Treatment for Candidiopotamon penglai

## ﻿Additional information

### Author ORCIDs

Hsi-Te Shih https://orcid.org/0000-0003-1317-8783

Tohru Naruse https://orcid.org/0000-0001-9212-5246

### Data availability

All of the data that support the findings of this study are available in the main text.
